# Modelling the significance of value-belief-norm framework to predict mass adoption potentials of internet of things-enabled wearable fitness devices

**DOI:** 10.1016/j.heliyon.2024.e30179

**Published:** 2024-04-28

**Authors:** Qing Yang, Abdullah Al Mamun, Mohammad Nurul Hassan Reza, Farzana Naznen

**Affiliations:** a UKM - Graduate School of Business, Universiti Kebangsaan Malaysia, 43600, UKM Bangi, Selangor Darul Ehsan, Malaysia; b UCSI Graduate Business School, UCSI University, Malaysia. No. 1, Jalan Menara Gading, UCSI Heights (Taman Connaught), Cheras, 56000, Kuala Lumpur, Malaysia

**Keywords:** IoT-enabled devices, Wearable fitness devices, Value-belief-norm model, Social norms, Young adults

## Abstract

Self-health monitoring technologies have become increasingly popular in averting unanticipated health complications. However, the adoption rate of such technologies in developing countries is surprisingly low. Furthermore, empirical studies on the application of the value-belief-norm (VBN) model to elucidate intention to use IoT-enabled wearable fitness devices (IoT-enabled WFDs) are scarce. This study aimed to expand the VBN model by integrating health values, health consciousness, health knowledge-seeking, and social norms as influencing constructs. The proposed holistic framework was empirically tested to examine these constructs on users' decision-making process of adopting IoT-enabled WFDs. A web-based survey involving 866 adults in China aged 18–30 years was conducted. The gathered data were analysed using partial least squares-structural equation modeling. The results revealed the significant influence of health consciousness and health knowledge-seeking on personal health beliefs, as well as the favourable impact of personal health beliefs on personal norms and awareness of consequences. The results further demonstrated the substantial influence of awareness of consequences and ascription of responsibilities on personal norms. Besides that, personal norms and societal norms were found to have strong influence on the intention to adopt IoT-enabled WFDs, which was revealed to have significant influence on the actual usage. This study's findings offer novel theoretical insights on the behavioural characteristics of adopting IoT-enabled WFDs and serve as a practical guideline for industry experts and marketers to establish appropriate marketing strategies to support the IoT-enabled wearable sector. The findings also benefit policymakers in their efforts of developing strategies that emphasise the unique benefits of self-healthcare monitoring to encourage active lifestyle and decrease obesity and overweight-related health risks.

## 1 Introduction

Obesity is a chronic health issue worldwide [1]. Ensuring active and healthy lifestyle is one of the most sensitive topics in society. Having people to live actively and healthily as they grow older is a delicate issue [2]. According to the US Health Department, the hazards of sedentary lifestyle and obesity in young people include type II diabetes, hypertension, heart disease, bone and joint difficulties, sleeping disorder, and cancers in the early age [3]. Obesity and health risks associated with unhealthy lifestyles can be effectively managed and addressed in young people by limiting unhealthy dietary habits, tobacco and alcohol consumption and encouraging physical activity [4], and the use of wearable fitness devices (WFDs) can help people to regularly monitor and record body data and health status, effectively providing early warning of obesity [5]. Regular physical exercise has been shown to reduce the risks of obesity, overweight, coronary heart disease, diabetes, high blood pressure, depression, and insomnia [6]. Thus, considering the role of health monitoring gadgets such as WFDs in daily physical activity, young people are becoming attracted and motivated to use such devices to understand their body's physical data, maintain their health and reduce the chances of obesity disease.

A growing market that introduces the capability of connecting multiple devices to provide real-time monitoring of health conditions has emerged [7]. In this context, wearable technologies have enabled on-body real-time scanning and computation of individual physiological parameters [8]. Wearable fitness trackers are the most extensively known and make use of Internet of Things (IoT)-enabled smart gadgets [9]. Studies have come up with the concept of wearable health technologies as a major resolution to obesity challenges [1,10]. Fitness trackers are defined as self-tracking devices that can be worn on the body, communicate with multi-sensor channels, including IoT [11], the current main user group is youth people and gather information of daily physical activities, workouts, and essential bodily parameters through trackers such as WFDs [12]. Numerous wearable fitness trackers have the capacity to allow users to fix their daily fitness targets and show progress towards those targets, enabling users to be held responsible to reach their everyday workout goals [9]. The worldwide IoT market reached USD 308.97 billion in 2020 [13], and the health and wellness wearable devices alone accounted for USD 275 million in 2021 [14]. The IoT-based wearable market in China was valued at more than 81 billion yuan in 2022. The market was predicted to grow to 93 billion yuan by 2023 [15]. Moreover, the revenue from the smartwatch industry is expected to rise significantly, reaching an astounding US$18.95 billion in 2024, according to forecasts. This expansion is anticipated to continue at a steady pace of 9.73 % each year, reaching a market volume of US$27.47 billion by 2028 [16]. Based on this huge market size, it is evident that the wearable medical device market has rapidly gained traction, with increasing numbers of young people paying attention to their own health status becoming potential user groups [5]. Although the WFD supporting IoT can bring more convenience and benefits to users, there is little research on the factors that affect the adoption intention and user acceptance of such technological devices, especially regarding the actual use of WFDs among adolescent populations [1,9]. Therefore, both industry experts and academics should gain a more comprehensive view by thoroughly investigating the behavioural and decision-making factors that affect users’ intention to adopt IoT-enabled WFDs.

This study observed a demographic gap in the existing literature on the adoption of IoT-enabled WFDs among young adults in China. Based on the Report on the Status of Nutrition and Chronic Diseases of the Chinese Population [17], which was released in a press conference held by the State Council Information Office on December 23, 2020, more than 50 % of Chinese adults were reported overweight or obese by the end of 2020. The overweight rate among Chinese adults has increased by about 20–40 %, as compared to the data in the Report on the Status of Nutrition and Chronic Diseases of the Chinese Population [17] released in 2015. The data comparison of the reports also showed an increase in the prevalence of hypertension, diabetes, chronic obstructive disease (COPD), and cancer prevalence from 2015 to 2020. Without an effective control, the figure is projected to further increase, posing a significant challenge to the prevention and control of chronic diseases. The high rate of obesity and accompanying health risks highlight the importance of regular health monitoring among young adults to provide essential care and treatments to prevent unpleasant incidence. Despite the fact that many young adults purchase and use fitness trackers, they remain in a high-risk category in terms of obesity and sedentary lifestyle [18]. In the existing literature, there is a dearth of thorough investigation on the young generation's intention to adopt IoT-enabled WFDs. Addressing this demographic gap, the current study targeted Chinese young adults of between 18 and 30 years old to evaluate the significant factors that influence their adoption of IoT-enabled WFDs for routine health monitoring. Most importantly, the current study identified several theoretical gaps in the literature on the adoption of IoT-enabled WFDs. Firstly, intrinsic variables that affect users' decision-making on IoT-enabled WFDs, such as health values, health consciousness, and health knowledge-seeking, have remained poorly theorised. Secondly, the close correlations of personal health beliefs and personal norms with users' intention to adopt IoT-enabled WFDs have remained relatively unexplored. Finally, there are limited empirical studies on the actual adoption of IoT-enabled WFDs based on the value-belief-norm (VBN) model. Nonetheless, this theory has the potential to analyse the adoption and integration of technology. It posits that individuals' decisions to adopt a specific technology are determined by three interconnected factors: values, beliefs, and norms. In the context of IoT-enabled WFDs, VBN theory helps to understand why individuals choose to adopt and use these devices. The theory suggests that people's values, beliefs, and norms shape their attitudes and intentions towards adopting new technologies, such as IoT-based wearables. Researchers and developers can benefit from integrating VBN theory into the design and implementation of IoT-based WFDs, as this allows them to create more effective behavioral interventions by utilizing theoretical insights. With that, this study aimed to bridge the gaps and achieve the research objectives by addressing the following research questions.

⁃ Do individuals' health values, consciousness and knowledge-seeking behaviour influence their health beliefs?
⁃ Do individuals' health beliefs affect their awareness of consequences and personal norms?
⁃ Does an individual's awareness of consequences influence their ascription of responsibilities and personal norms?
⁃ Does an individual's ascription of responsibility impact their personal norms?
⁃ Do individuals' personal and social norms influence their intention to adopt IoT-enabled WFDs?
⁃ Does an individual's intention to adopt IoT-enabled WFDs influence their actual adoption of these devices?

The extended model was then empirically tested to explore users' decision-making process to adopt IoT-enabled WFDs. The findings of this study offer useful insights that benefit policymakers, industry experts, healthcare institutions, and IT developers to develop successful promotional strategies that emphasise the benefits of IoT-enabled WFDs. These strategies aim to enhance users’ attitude and intention to adopt these technologies by instilling confidence in them. The findings particularly benefit healthcare providers in recognizing the long-term effectiveness of IoT-enabled WFDs to enhance community healthcare services.

## 2 Literature review

### 2.1 Historical development of WFDs and IoT technology

The evolution of WFDs and IoT technology is a compelling story of innovation, fueled by breakthroughs in electronics, miniaturization, and the increasing importance of personal health and fitness. The history of WFDs can be traced back to early mechanical pedometers which were primarily used for counting steps. In the 1980s and 1990s, digital pedometers and heart rate monitors became popular among fitness enthusiasts and athletes, offering improved accuracy and data storage and analysis capabilities [19]. The convergence of wearable technology with the IoT marked a transformative moment, enabling wearables to connect to the internet and share data seamlessly, leading to new possibilities for remote monitoring, data synchronization, and real-time feedback [20]. In the mid-2000s, smartwatches and fitness bands emerged, combining fitness tracking features with smartphone connectivity to allow users to receive notifications, control apps, and track various health metrics simultaneously. Advanced sensors, including accelerometers, gyroscopes, heart rate monitors, and GPS, further enhanced the capabilities of wearables. These sensors enabled precise tracking of activities such as steps taken, distance travelled, sleep patterns, and heart rate variability [21]. The evolution of wearable technology has expanded beyond fitness tracking to encompass broader health and medical applications, including monitoring chronic conditions, early disease detection, and remote patient care. Wearables are now positioned as valuable tools in the healthcare ecosystem. Looking ahead, the future of IoT-enabled wearables appears promising, with ongoing innovations in materials, power efficiency, and connectivity protocols likely to result in even greater integration into daily life.

### 2.2 Theoretical background

The theory of reasoned action (TRA) and theory of planned behaviour (TPB) are widely used to comprehend various health activities. Researchers have employed these theories to better understand physical activities [22], blood pressure monitoring [23], and blood oxygen status [24]. Although the TRA provides a strong foundation for comprehending user intentions, it may not consider the broader context and societal factors that play an increasingly critical role in today's interconnected society [25]. While TPB improves the accuracy of intention-based models, it may not be sufficient to fully understand the intricate relationship between individual and societal factors that influence technology adoption [26]. Studies have also attempted to combine and expand unified theory of acceptance and use of technology (UTAUT), technology acceptance model (TAM), and TRA, to elucidate the adoption of information technology in healthcare and related health monitoring devices [12,[27], [28], [29]]. Moreover, TAM provides valuable insights into user acceptance, but has been criticized for its narrow focus on cognitive factors, as it tends to overlook the social and normative aspects that can influence technology adoption [30]. Although UTAUT provides a comprehensive framework, it may not fully explore intrinsic factors, such as value systems, personal beliefs, and societal norms, that influence adoption behaviours [31]. However, only a few prior studies included norm activation theory (NAT) in conjunction with other theories to study the adoption of healthcare practices and personal health monitoring devices [24,32].

To the best of the researchers' knowledge, the VBN model has yet to be fully adopted to empirically examine users' decision-making process regarding fitness monitoring devices. To fill these gaps, the VBN theory's inclusion of values and societal norms broadens the explanatory scope to encompass a wider range of influences on adoption decisions. This study also incorporated personal norms into the traditional VBN theory, representing a significant expansion that deepens our understanding of the impact of social norms and expectations on behavior. The VBN theory primarily concentrates on the role of social norms in shaping individual behavior. However, the idea of personal norms introduces an extra layer by considering internalized beliefs and self-imposed standards that direct an individual's actions. Moreover, we have integrated an understudied construct, namely health knowledge-seeking, which enriches existing theoretical frameworks by offering a more comprehensive understanding of the cognitive processes and motivational factors that underpin technology adoption and health behaviours. Incorporating this construct facilitates a nuanced understanding of individuals' information-seeking behaviours and the role that health literacy plays in shaping adoption decisions. Thus, by incorporating values, beliefs, and norms, the VBN theory expands our understanding of why individuals opt to use WFDs, providing a comprehensive perspective that is consistent with the intricate and multi-dimensional nature of technology adoption in modern society.

The VBN theory, developed by Stern et al. [33] offers a comprehensive perspective for analysing the adoption and integration of technology. It posits that individuals' decisions to adopt a specific technology is determined by three interconnected factors: Value, Belief, and Norm. The “Value” component of the VBN theory posits that individuals are more likely to adopt a technology when they perceive it as valuable for achieving their personal goals and objectives [33]. In the context of IoT-enabled WFDs, users may be motivated by the perceived value of these devices, such as improved health, fitness, convenience, and data-driven decision-making about their physical activity. The “Belief” component of the VBN theory refers to an individual's beliefs regarding the effectiveness of a technology [33]. The likelihood of users adopting a technology increase if they believe it can help them achieve their desired outcomes. In the case of WFDs, users may adopt these devices due to their belief that monitoring their activities and receiving feedback through the device will result in positive health and fitness outcomes. The “Norm” component of VBN theory examines the impact of societal norms and expectations on technology adoption [33]. It suggests that individuals are more likely to adopt a technology if it conforms to prevailing social norms. In the context of WFDs, norms regarding health-consciousness, technology-aided self-improvement, and peer behaviour may significantly influence adoption decisions. These norms can shape users' perceptions of the social acceptability and appeal of using these devices. Individual elements of the VBN model, such as health interest [34], personal health values [35], health beliefs [36], health information-seeking [1], awareness of consequences [24], ascription of responsibility [37], and moral consideration [38], have been studied in isolation. This study extending the model by incorporating additional constructs to empirically analyse the adoption of IoT-enabled WFDs among young adults.

### 2.3 Hypotheses development

#### 2.3.1 Health values (HV)

As part of personal values, health is linked to one's proactive activity to improve the current health status and future health status [39]. The perception of health values can be thoroughly analysed by the degree to which an individual believes that good health is the most essential aspect of having a happy life [40]. These values reflect individuals' intrinsic beliefs and priorities regarding the importance of maintaining good health and well-being. Thus, they perceive that “nothing is more important than good health”, reflecting their overarching beliefs about the fundamental importance of health in their lives [40]. Individuals with high perceived health values demonstrate a strong sense of their own healthcare, their concern about their personal health issues, and their beliefs that taking healthcare precautions and measures can prevent the consequences of poor health [41]. Individuals who value their health make use of self-tracking devices to assess their health conscientiously and explicitly (in terms of pulse rate, sleeping time, daily exercise, calorie burns, somatic symptoms, anxiety, and recovery) for them to evaluate and enhance their health fitness accordingly [42]. The higher one's perceived health values, the higher one's health beliefs and confidence in the ability to properly manage the health status in the face of any associated health risks [36]. In view of the above, the following hypothesis was proposed.

H1 Health values positively influence personal health beliefs.

#### 2.3.2 Health consciousness (HC)

Health consciousness reflects an individual's perspectives towards health issues and readiness to take measures to protect health [12]. It also refers to an individual's beliefs towards the progression in personal health conditions and the importance placed on healthy practices [43]. Zhang et al. [32] empirically demonstrated the inclination of using health-related technologies when people are concerned about their health issues. Health-conscious individuals are particularly worried about declining health conditions, which drive them to believe that taking measures to enhance and/or protect their health can improve their physical condition. Thus, they believe that “living a healthy life is important to me”, capturing their conscious efforts to prioritize health and integrate healthy practices into their daily lives [12]. Individuals with higher health consciousness are more likely to believe that a WFD can provide stronger inspirational environment for them to engage in fitness activities [44]. Based on the preceding arguments, the following hypothesis was posited.

H2 Health consciousness positively influences personal health beliefs.

#### 2.3.3 Health knowledge-seeking (HKS)

Individuals who seek out health-related information are referred to as “health information seekers”; for instance, when people realise that they are overweight, they would seek out knowledge to reduce the risk of obesity [1]. Earlier studies revealed a strong relationship between personal health beliefs and the propensity to adopt online health information systems [35,45]. Individuals with strong health beliefs actively search for ways to enhance their health and are more inclined to learn about the usefulness of wearable health device, resulting in the sense of having greater utility to improve their present health state [32]. Health information seekers who believe that their inactivity may cause obesity are more likely to use a wearable activity-tracking device to monitor their food consumption and physical activity levels [1]. Based on the rationale of the existing literature, the following hypothesis was outlined.

H3 Health knowledge-seeking positively influences personal health beliefs.

#### 2.3.4 Personal health beliefs (PHB)

“Health beliefs” refer to one's views about the feasibility of particular activities in enhancing one's health state [32]. Health beliefs postulates higher inclination to make decisions concerning one's personal fitness practices due to the awareness of the adverse consequences of an inactive lifestyle and beliefs that health practices can facilitate and improve the overall health and well-being [46]. A high level of health beliefs in the usability of healthcare wearable devices promotes awareness of the advantages and implications of the devices in health management, which encourages the intention to adopt such devices [29]. When individuals express higher level of health beliefs and actively seek innovative ways to enhance the health status, they are more likely to gain higher level of awareness regarding the utility of wearable healthcare devices [28]. This may develop their personal norms of using these devices. Based on the potential features of wearable health technologies, numerous empirical studies have employed health beliefs as a key construct to evaluate users' intention to adopt wearable healthcare and fitness monitoring technologies [28,32]. Therefore, the following hypotheses were proposed.

H4a Personal health beliefs positively influence awareness of consequences.

H4b Personal health beliefs positively influence personal norms.

#### 2.3.5 Awareness of consequences (AOC)

The understanding that one's activities influence the well-being of others is recognized as awareness of consequences [46]. It activates personal norms [47] and is closely related to the ascription of responsibility [48]. The awareness of various ailments depends on the increase in self-management of health, which requires one to be more educated about the diseases and responsible for the adverse consequences [35]. Individuals who are keenly aware of the consequences of chronic diseases may share information with their trusted others and suggest taking the necessary health precautions, such as the use of fitness and health self-monitoring technologies, in order to reduce the feeling of hopelessness and anxiety of the adverse consequences [27]. Shanka and Gebremariam [24] empirically established awareness of the adverse consequences of failing to comply with the COVID-19 safety standards as a crucial driver of moral and practical changes. Based on the discussion, the following hypotheses were developed.

H5a Awareness of consequences positively influences ascription of responsibility.

H5b Awareness of consequences positively influences personal norms.

#### 2.3.6 Ascription of responsibility (AOR)

Ascription of responsibility refers to the process of accepting accountability for the performed activities [49]. Individuals who realise the consequences of their activities and are prepared to be responsible for these activities are more likely to have their personal norms triggered [50]. The assignment of responsibility to oneself is a prerequisite for developing personal norms, which subsequently influences one's health safety compliance behaviour [24]. Sharon [37] recommended developing a strong sense of personal responsibility to care one's own health and fitness to boost the general health of the community. Furthermore, the experience with self-monitoring technologies can be characterised by improved insights and understanding of the associated health hazards, a stronger sense of control over health fitness, and an increased sense of personal accountability [23]. Given the significance of ascription of responsibility in the formation of personal norms within the context of fitness monitoring, the following hypothesis was postulated.

H6 Ascription of responsibility positively influences personal norms.

#### 2.3.7 Personal norms (PN)

Personal norms refer to one's characteristics of realisation that one's actions may result in favourable or unfavourable outcomes for others and the preparation to act accordingly out of their internalized values [50]. The adoption of an AI system in healthcare requires users' evaluation from the moral perspectives [51], which strongly justifies the significance of personal norms in fitness monitoring. Physical slothfulness in the condition of overweight or obesity is liable to personal moral judgment in a community, where participating in physical exercises is acknowledged as moral norms [52]. Shanka and Gebremariam [24] observed the major influence of strong personal norms on the compliance with COVID-19 preventative measures. The above discussion suggests that strong personal norms may enhance individual's willingness to use fitness monitoring devices, which led to the development of the following hypothesis.

H7 Personal norms positively influence the intention to adopt IoT-enabled WFDs.

#### 2.3.8 Social norms

Social norms refer to an individual's judgments of the suggestions and viewpoints from other influential individuals around them on whether or not they should engage in a certain activity [29]. For instance, certain individuals are more likely to embrace wearable healthcare devices if a famous and prominent figure recommends using these devices [29]. Considering the increasing societal pressures and fitness trends across social media platforms, the influence of social norms within the context of fitness trackers is especially noteworthy [53]. Social norms are typically thought to be external factors stemming from societal expectations, cultural norms, and peer influences. While social norms and values/beliefs are interrelated and can influence each other, they are distinct components of an individual's cognitive processing and behavioral influence. In this study, social norms are considered a single construct that affects individuals' intention to adopt IoT-enabled WFDs, highlighting the significance of external social influences in shaping adoption decisions. This perspective aligns with the existing literature [54], where researchers have also explored social norms as a standalone construct in their theoretical frameworks, acknowledging their unique impact on behavioral intentions. Individuals with positive health enhancement experience of using wearable healthcare technologies are more likely to recommend these technologies to their acquaintances and encourage others to become consistent users based on their social acceptance [29]. Reith et al. [53] found that individuals tend to assimilate the recommendations of key individuals around them before they decide to use fitness trackers and believe that fitness trackers can truly improve their physical activity performance. In a more recent study, Clubbs et al. [6] empirically demonstrated the major influence of social norms on users' intention to use wearable fitness trackers. Based on the findings of prior studies, this study proposed the following hypothesis.

H8 Social norms positively influence the intention to adopt IoT-enabled WFDs.

#### 2.3.9 Intention to adopt IoT-Enabled WFDs and actual adoption

Individuals' behaviour can be characterised as an intentional activity that is carried out as a result of numerous factors [55]. One's intention influences the resultant behaviour, as individuals tend to first think logically and subsequently intend to achieve a certain objective. According to Ahmed et al. [56] the materialistic intention of using IoT exhibits considerable influence on consumers' actual usage of IoT. Users' intention of using e-Health devices determine the actual usage of health monitoring technology [38]. Although wearable healthcare devices possess many common characteristics with other technology-related products and services, the usage of these devices is associated with a sensitive context (interpretation of physical health state); therefore, it is critical to thoroughly investigate users' intention to accept these technologies and their actual adoption of these technologies [29]. In view of the above, the following hypothesis was proposed.

H9 Intention to adopt IoT-enabled WFDs positively influences their actual adoption.

**Fig. 1:**
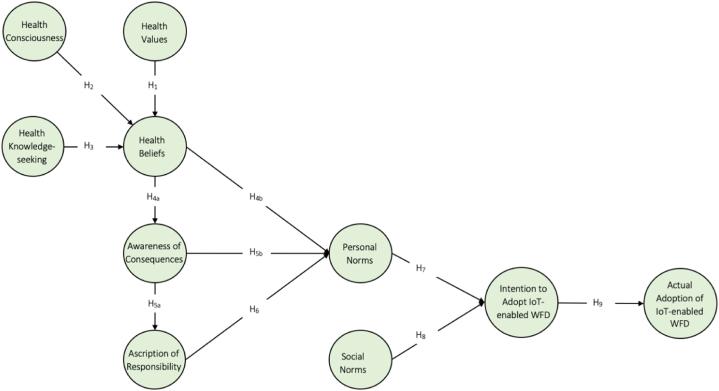
Research framework.

All the associations hypothesized above are presented in Fig. 1.

## 3 Methodology

This study applied a cross-sectional quantitative method to examine the relationships between the constructs. Partial least squares-structural equation modeling (PLS-SEM) was performed using the SmartPLS (V.3.3.5) software. As PLS-SEM is a non-parametric and multivariate approach, it is widely used to evaluate path correlations with latent variables [57]. Furthermore, PLS-SEM is recommended for exploratory research when the research framework is highly complicated and involves mediating or moderating variables [58]. The nature of the current study was exploratory, which included many independent variables at different levels and aimed to evaluate both direct and indirect effects of multiple factors. Thus, PLS-SEM was deemed appropriate for data analysis in this study.

### 3.1 Population and sample size

The population of this study was young adults aged 18–30 years, who are known for their keen interest in and adoption of technology, including WFDs. This age group has been found to be more likely to try out new technologies and adopt those that enhance their overall well-being. Therefore, targeting this younger demographic is crucial for the development and marketing of technology-driven health and fitness solutions. The 18–30 age group is particularly tech-savvy, health-conscious, and open to incorporating digital tools into their daily routine. Given the recent surge in the availability of IoT-enabled WFDs, it is essential to understand the perspectives and behaviours of young adults regarding these technologies. This knowledge can be used to predict future trends, shape product development strategies, and encourage innovation in the wearable-fitness industry.

Convenience sampling strategy was employed in this study. The primary reason for selecting convenience sampling was the ease of access to potential participants [59]. Given the prevalence of individuals who used WFDs, convenience sampling enabled us to quickly and efficiently reach a large pool of respondents who met our inclusion criteria. Convenience sampling is a cost-effective method for collecting data [60]. Thus, by targeting readily available participants from existing networks or communities, we minimized the costs associated with participant recruitment, travel, and other logistical expenses. Convenience sampling also allowed us to expedite the data collection process, ensuring timely completion of the study within the allotted schedule [61]. Furthermore, sample homogeneity was not a primary concern in this study. Therefore, to understand the adoption of IoT-enabled WFDs within a specific demographic or user group, convenience sampling provided an appropriate approach for targeting individuals who met our criteria for participation. Hair Jr et al. [57] suggested a sample size of at least 200 for the SEM. As recommended by Faul et al. [62], the G*power tool was used to calculate the exact minimum sample size correctly. The results showed that this study required at least 166 respondents based on the following parameters: effect size (*f*^2^) of 0.15, *α* err prob of 0.05, power (1 − *β* err prob) of 0.95, and nine predictors. Additionally, the estimated sample size was 550, according to the formula *n* = 100 + 50*i*, as previously established by Bujang et al. [63], to determine the appropriate sample size based on the number of independent variables (*i*) in the framework. However, to ensure a comprehensive analysis, we decided to collect data more than the estimated sample size of 550. Moreover, the complexity of the current research model, encompassing the number of latent variables, observed indicators, and structural paths, was considered when selecting the sample size. Although larger sample sizes are typically preferred for more intricate models with numerous parameters, PLS-SEM analysis is recognized for its capacity to handle intricate models with smaller sample sizes. Thus, a sample size of 866 respondents was sufficient to capture the variability in the data and estimate the model parameters with acceptable levels of accuracy and reliability.

### 3.2 Data collection

An online survey was conducted to collect data. A series of questionnaire sets were distributed via WJX online link. The online survey was mainly conducted through online channel platforms, such as WeChat and QQ. Before data collection, ethical approval was obtained from the Ethics Committee of Changzhi University. In order to fully protect the personal privacy and information security of the participants, all participants were informed of the purpose and data flow of this study, as well as their right to withdraw from the study at any time before and during filling out the questionnaire. Based on this, all participants provided online informed consent. All data were randomly obtained from 866 respondents to reduce the complexities of small sample size.

### 3.3 Measurement items

The questionnaire was designed using previously validated instruments with slight modifications to fit the context of the current study. To enhance the validity of the responses and preserve the instrument's integrity, language modifications were made to ensure cultural appropriateness and understanding among the participants. These adjustments were aimed at improving the accuracy of the responses and maintaining the validity of the research findings. The development of the questionnaire ensured the use of unambiguous, simple, and unbiased phrasing to maintain the interest and concentration of the respondents to provide accurate views and responses with ease. We also made minor modifications to the language of certain items to ensure clarity and relevance to our specific study population. These adjustments were made while preserving intended meaning and construct validity. The questionnaire was initially created in English; however, as the targeted respondents were in China, it was translated into Chinese by a professional translator. To avoid significant cross-cultural differences, five individuals proficient in both Chinese and English were selected to perform a pre-test. Based on their feedback, some wording was modified. Finally, to ensure the validity and reliability of the questionnaire, 40 samples were collected for a pilot test before the final data collection.

Regarding the measurements, four items derived from Lau et al. [40] were used to measure health values. Five items of health consciousness were adapted from Yang et al. [12], while five items of health knowledge-seeking were adapted from Hong [64]. Five items were integrated from Kraft and Goodell [65] to measure health beliefs. Awareness of consequences and ascription of responsibility were assessed using five items of each from Stern et al. [33]. Another five items were adapted from Yang et al. [66] to measure personal norms. Items of social norms were adapted from Doran and Larsen [67]. Besides that, the intention to adopt IoT-enabled WFDs was assessed using five items derived from Alam et al. [68] and Wang et al. [69] Finally, one item was included to measure the actual usage of IoT-enabled WFDs. The respondents were required to provide their views and responses on a seven-point Likert scale. All items are presented in Supporting Material S1. Table S1. *Survey Instrument*.

## 4 Results

### 4.1 Demographic characteristics of respondents

**Fig. 2:**
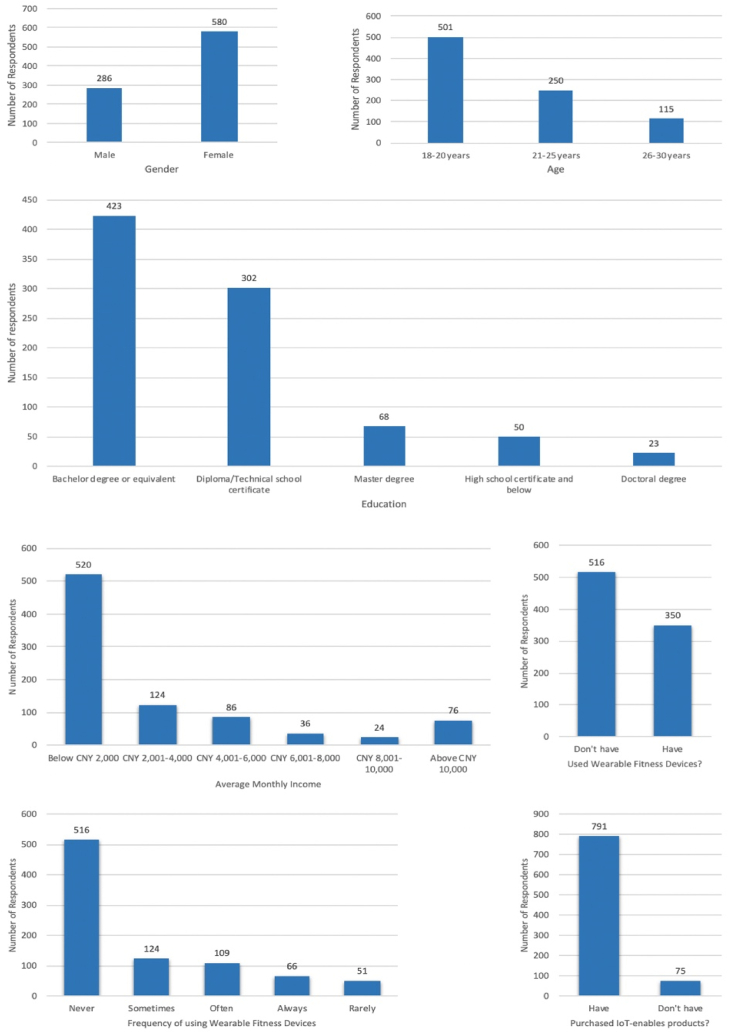
Demographic Details. Note: The horizontal axis shows the respondents' demographics, while the vertical axis shows the number of respondents.

The respondents' demographic characteristics are demonstrated in Fig. 2. Referring to the figure, most of the respondents (580) were female, and the remaining (286) were male. The majority of the respondents (423) had a Bachelor's degree. More than half of the respondents (501) were between the ages of 18 and 21. Regarding the monthly income, 520 respondents reported earning lower than CNY 2000 per month. Only 350 respondents reported using WFD, although 791 respondents indicated that they purchased other IoT-enabled devices.

### 4.2 Common method bias

Several measures were taken to prevent common method bias from affecting data. At the outset of the data collection process, clear and explicit instructions were provided at the beginning of the questionnaire. Moreover, respondents were apprised that there were no correct or incorrect answers, thereby fostering a candid and open-response environment. Furthermore, we ensured confidentiality and anonymity of the respondents by refraining from collecting any personal identifiers. All initiatives were implemented to mitigate the issue of common method bias.

We also employed Harman's single-factor test to assess the presence of the common method variance issue to confirm that the study's model is not significantly influenced by common method bias [70]. The single component explained 46.58 % of total variation, which did not exceed the 50 % criterion proposed by Podsakoff et al. [71]. In other words, the common method bias was not an issue in the current study. As recommended by Kock [72], a full collinearity test (Table 1) was performed on all constructs, which revealed that all values of variance inflation factor (VIF) (ranging from 1.064 to 4.253) did not exceed the maximum threshold value of 5 [72]. These results reaffirmed that common method bias was not an issue in this study.

**Table 1:** Full collinearity test.

Variables	VIF
Health Values	2.211
Health Consciousness	2.795
Health Knowledge-seeking	3.114
Health Beliefs	2.413
Awareness of Consequences	2.179
Ascription of Responsibility	2.674
Personal Norms	4.253
Social Norms	2.735
Intention to Adopt IoT-enabled WFD	2.984
Actual Adoption of IoT-enabled WFD	1.064

### 4.3 Measurement model (Outer model)

The measurement model in this study was evaluated in terms of internal consistency, convergent validity, and discriminant validity [58].

#### 4.3.1 Internal consistency and convergent validity

Internal consistency and convergent validity of the constructs were assessed based on Dijkstra-Hensele's *rho*, Cronbach's alpha, and composite reliability; values greater than 0.70 indicate strong internal consistency and reliability [73]. As shown in Table 2, Cronbach's alpha (values ranging from 0.836 to 1.000), Dijkstra-Hensele's *rho* (values ranging from 0.869 to 1.000), and composite reliability (values ranging from 0.886 to 1.000) exceeded the threshold value of 0.7. These results validated the model's strong reliability and internal consistency. Besides that, the values of AVE were used to evaluate convergent validity; AVE reflects how much variance in the respective construct that the latent constructs explain [58]. Hair et al. [57] recommended that the values of AVE should exceed 0.5 to ensure that the model and its components exhibit significant convergent validity. Referring to Table 2, the AVE values range from 0.614 to 1.000, indicating high convergent validity.

**Table 2:** Validity and reliability.

Variables	Cronbach's Alpha	Dijkstra-Hensele's *rho*	Composite Reliability	Average Variance Extracted
Health Values	0.920	0.924	0.944	0.808
Health Consciousness	0.927	0.929	0.945	0.775
Health Knowledge-seeking	0.905	0.919	0.930	0.726
Health Beliefs	0.836	0.869	0.886	0.614
Awareness of Consequences	0.946	0.950	0.959	0.824
Ascription of Responsibility	0.959	0.960	0.968	0.860
Personal Norms	0.966	0.966	0.973	0.880
Social Norms	0.941	0.946	0.955	0.810
Intention to Adopt IoT-enabled WFD	0.939	0.940	0.954	0.806
Actual Adoption of IoT-enabled WFD	1.000	1.000	1.000	1.000

#### 4.3.2 Discriminant validity

To determine the discriminant validity of the model, the Fornell-Larcker criteria and Heterotrait-Monotrait (HTMT) ratio were utilised. The square root values of AVE should exceed the variances of any other latent variables when it comes to evaluating the Fornell-Larcker criteria [58]. As shown in Table 3, the values of the Fornell-Larcker criteria exceeded the recorded correlations. Meanwhile, according to Henseler and Sarstedt [74], all HTMT values should not exceed 0.85 to attain significant discriminant validity. Referring to Table 3, all HTMT values ranged from 0.051 to 0.817, which did not exceed the threshold value.

**Table 3:** Discriminant validity.

	HV	HC	HKS	PHB	AOC	AOR	PNS	SNS	IWFD	AWFD
*Fornell-Larcker Criterion*										
HV	0.899									
HC	0.728	0.880								
HKS	0.481	0.572	0.852							
PHB	0.524	0.632	0.686	0.784						
AOC	0.511	0.589	0.514	0.609	0.907					
AOR	0.561	0.637	0.640	0.647	0.669	0.927				
PNS	0.403	0.471	0.754	0.617	0.419	0.510	0.900			
SNS	0.305	0.352	0.592	0.433	0.401	0.344	0.752	0.898		
IWFD	0.405	0.461	0.680	0.567	0.410	0.528	0.776	0.683	0.938	
AWFD	0.089	0.057	0.128	0.077	0.050	0.065	0.166	0.163	0.227	1.000
*Heterotrait-Monotrait Ratio (HTMT)*										
HV	–									
HC	0.786	–								
HKS	0.511	0.611	–							
PHB	0.569	0.687	0.790	–						
AOC	0.546	0.625	0.530	0.640	–					
AOR	0.596	0.674	0.664	0.687	0.702	–				
PNS	0.429	0.501	0.817	0.707	0.443	0.531	–			
SNS	0.327	0.377	0.648	0.503	0.428	0.361	0.803	–		
IWFD	0.429	0.487	0.723	0.636	0.428	0.548	0.811	0.715	–	
AWFD	0.093	0.059	0.135	0.092	0.051	0.066	0.172	0.167	0.231	–

**Note:** HV: Health Values, HC: Health Consciousness, HKS: Health Knowledge-Seeking, PHB: Health Beliefs, AOC: Awareness of Consequences, AOR: Ascription of Responsibility, PNS: Personal Norms; SNS: Social Norms, IWFD: Intention to Adopt IoT-enabled WFD, AWFD: Actual Adoption of IoT-enabled WFD.

### 4.4 Structural model (Inner model)

To assess the structural model (as presented in Supporting *Material S1.* Figure S1*. PLS-SEM Model*), Hair et al. [58] recommended using path coefficient (beta value, *β*), coefficient of determination (*R*^2^), effect size (*f*^2^), and predictive relevance (*Q*^2^). To test the hypotheses, this study performed bootstrapping to calculate the p-values, t-values, and path coefficients. Finally, the indirect effects of the respective constructs were examined.

#### 4.4.1 Hypothesis testing

**Fig. 3:**
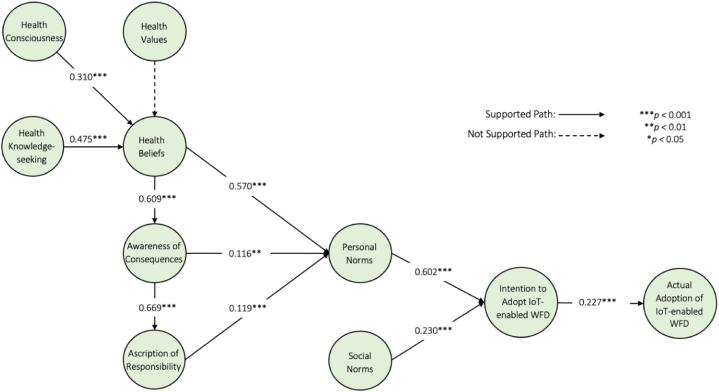
Final model and findings.

As shown in Fig. 3 and Table 4, health values in this study had no significant influence on personal health beliefs (*β* = 0.070, *t* = 1.588, *p* = 0.056). In other words, H1 was rejected. The results demonstrated significant and positive effects of health consciousness (*β* = 0.310, *t* = 6.168, *p* = 0.000) and health knowledge-seeking (*β* = 0.475, *t* = 12.640, *p* = 0.000) on personal health beliefs. Thus, H2 and H3 were supported. In addition, the results revealed the substantial and positive influence of personal health beliefs on both awareness of consequences (*β* = 0.609, *t* = 17.280, *p* = 0.000) and personal norms (*β* = 0.570, *t* = 17.462, *p* = 0.000), confirming H4a and H4b. Similarly, awareness of consequences was found to exhibit strong influence on ascription of responsibility (*β* = 0.669, *t* = 18.398, *p* = 0.000) and personal norms (*β* = 0.116, *t* = 3.110, *p* = 0.001). Thus, H5a and H5b were supported. Likewise, ascription of responsibility exhibited significant influence on personal norms (*β* = 0.199, *t* = 4.142, *p* = 0.000), supporting H6. Additionally, personal norms (*β* = 0.602, *t* = 11.911, *p* = 0.000) and social norms (*β* = 0.230, *t* = 4.383, *p* = 0.000) were found to have substantial positive influence on the intention to adopt IoT-enabled WFDs. Therefore, H7 and H8 were accepted. Finally, the results revealed a strong positive correlation between the intention to adopt IoT-enabled WFDs and actual adoption of IoT-enabled WFDs (*β* = 0.227, *t* = 7.662, *p* = 0.000), which supported H9.

**Table 4:** Hypothesis testing.

Hypo	Direct Effects	Beta	*t value*	*p value*	Decision
H_1_	HV → PHB	0.070	1.588	0.056	Rejected
H_2_	HC → PHB	0.310	6.168	0.000	Supported
H_3_	HKS → PHB	0.475	12.640	0.000	Supported
H_4a_	PHB → AOC	0.609	17.280	0.000	Supported
H_5a_	AOC → AOR	0.669	18.398	0.000	Supported
H_4b_	PHB → PNS	0.570	17.462	0.000	Supported
H_5b_	AOC → PNS	0.116	3.110	0.001	Supported
H_6_	AOR → PNS	0.199	4.142	0.000	Supported
H_7_	PNS → IWFD	0.602	11.911	0.000	Supported
H_8_	SNS → IWFD	0.230	4.383	0.000	Supported
H_9_	IWFD → AWFD	0.227	7.662	0.000	Supported

**Note:** HV: Health Values, HC: Health Consciousness, HKS: Health Knowledge-Seeking, PHB: Health Beliefs, AOC: Awareness of Consequences, AOR: Ascription of Responsibility, PNS: Personal Norms; SNS: Social Norms, IWFD: Intention to Adopt IoT-enabled WFD, AWFD: Actual Adoption of IoT-enabled WFD.

#### 4.4.2 Coefficient of determination (R^2^)

The coefficient of determination (*R*^2^) denotes the degree of explained variance, referring to the proportion of the variation in the dependent variable explained in a linear model. Endogenous latent variables with *R*^2^ of 0.75, 0.50, or 0.25 are considered substantial, moderate, or weak, respectively [57]. All values of *R*^2^ for endogenous variables are tabulated in Table 5. Based on the recorded *R*^2^ value of personal health beliefs (0.400), the exogenous variables, namely health value, health consciousness, and health knowledge-seeking, explained 40.0 % of total variation in personal health beliefs, indicating moderate explanatory power. Similarly, the results of the remaining constructs suggested moderate to poor explanatory power: personal norms (55.7 %); awareness of consequences (37.0 %); ascription of responsibility (44.7 %); intention to adopt IoT-enabled WFDs (62.4 %); actual adoption of IoT-enabled WFDs (5.1 %).

**Table 5:** Coefficient of determination.

Variables	R Square	R Square Adjusted	Explanatory power
Personal Health Beliefs	0.402	0.400	Moderate
Personal Norms	0.558	0.557	Moderate
Awareness of Consequences	0.371	0.370	Weak
Ascription of Responsibility	0.448	0.447	Weak
Intention to IOT enabled Adopt WHDs	0.625	0.624	Moderate
Actual Adoption of IOT enabled WHDs	0.052	0.051	Weak

Note: R^2^ value interpretation ( ≥ 0.75- Significant, ≥ 0.50- moderate, ≥ 0.25- Weak)^49^.

#### 4.4.3 Effect size (f^2^)

Effect size (*f*^2^) measures the substantial influence of exogenous factors on endogenous variables based on the particular variance, instead of their shared variance [58]. Cohen [75] classified the effect size as trivial (<0.02), minor (≥0.02), medium (≥0.15), and substantial (≥0.35). However, it may be difficult to guarantee that these rules of thumb are adequate to produce large effect size since the outcomes may vary, depending on the characteristics of the research framework and domain of interest [58]. Table 6 presents the results of effect size. Personal health beliefs had a substantial effect on awareness of consequences, and awareness of consequences had a substantial effect on ascription of responsibility. Meanwhile, health values had a trivial effect on personal health beliefs, whereas awareness of consequences had trivial effect on personal norms. The results further revealed the medium effect of health knowledge-seeking on personal health beliefs, as well as the medium effect of personal health beliefs on personal norms. Finally, the remaining constructs had small effects on the corresponding endogenous variables.

**Table 6:** Effect size (*f*^*2*^*)*.

Associations	*f*^*2*^	Effect Size
HV→PHB	0.005	Trivial
HC→PHB	0.088	Small
HKS→PHB	0.340	Medium
PHB→AOC	0.589	Substantial
AOC→AOR	0.812	Substantial
PHB→PNS	0.219	Medium
AOC→PNS	0.000	Trivial
AOR→PNS	0.030	Small
PNS→IWFD	0.420	Substantial
SNS→IWFD	0.061	Small
IWFD→AWFD	0.054	Small

Note1: HV: Health Values, HC: Health Consciousness, HKS: Health Knowledge-Seeking, PHB: Health Beliefs, AOC: Awareness of Consequences, AOR: Ascription of Responsibility, PNS: Personal Norms; SNS: Social Norms, PPV: Perceived Product Value, IWFD: Intention to Adopt IoT-enabled WFD, AWFD: Actual Adoption of IoT-enabled WFD.

Note2: f^2^ score interpretation ( ≥ 0.35- substantial effect size, ≥ 0.15– medium effect size, ≥ 0.02- small effect size and <0.02- trivial effect size) [75].

#### 4.4.4 Predictive relevance (Q^2^)

The predictive relevance (*Q*^2^) determines whether the exogenous factors outperform the endogenous variables in terms of predictive power [58]. In order to have the predictive relevance of exogenous variables on the endogenous variables, the value of *Q*^2^ must exceed zero [58]. The results presented in Table 7 indicated that all endogenous variables in this study attained *Q*^2^ values of more than zero, indicating the strong predictive power and validity of the model.

**Table 7:** Predictive relevance (*Q*^*2*^*)*.

Variables	SSO	SSE	Q^2^(=1-SSE/SSO)
Health Beliefs	4330.000	2867.422	0.338
Personal Norms	4330.000	2942.184	0.321
Awareness of Consequences	4330.000	3024.050	0.302
Ascription of Responsibility	4330.000	2676.000	0.382
Intention to Adopt IoT-enabled WFD	4330.000	1966.017	0.546
Actual Adoption of IoT-enabled WFD	866.000	824.284	0.048

Note: Q^2^ > 0 is significant.

#### 4.4.5 Indirect effects

Apart from the direct relationships between constructs, indirect effects should be evaluated to truly comprehend the relationships of the constructs [73]. Table 8 presents the obtained results on the indirect effects of all constructs. The results revealed significant and positive indirect effects of health consciousness and health knowledge-seeking on awareness of consequences, ascription of responsibility, personal norms, intention to adopt IoT-enabled WFDs, and actual adoption of IoT-enabled WFDs. In addition, this study found that the indirect effects of personal health beliefs on personal norms, ascription of responsibility, intention to adopt IoT-enabled WFDs, and actual adoption of IoT-enabled WFDs were statistically significant and positive. The results further revealed significant and positive indirect effects of awareness of consequences on personal norms, intention to adopt IoT-enabled WFDs, and actual adoption of IoT-enabled WFDs. Finally, the indirect effects of ascription of responsibility, personal norms, and social norms on actual adoption of IoT-enabled WFDs were statistically significant and positive.

**Table 8:** Indirect effects.

	Beta	*t Value*	*p value*
HV→AOC	0.042	1.583	0.057
HV→AOR	0.028	1.548	0.061
HV→PNS	0.040	1.593	0.056
HV→IWFD	0.024	1.579	0.057
HV→AWFD	0.005	1.563	0.059
HC→ AOC	0.189	5.676	0.000
HC→AOR	0.126	5.101	0.000
HC→PNS	0.177	6.256	0.000
HC→IWFD	0.106	5.631	0.000
HC→AWFD	0.024	4.707	0.000
HKS→AOC	0.289	10.067	0.000
HKS→AOR	0.194	7.742	0.000
HKS→PNS	0.271	8.442	0.000
HKS→IWFD	0.163	6.542	0.000
HKS→AWFD	0.037	5.130	0.000
PHB→PNS	0.070	2.953	0.002
PHB→AOR	0.407	9.724	0.000
PHB→IWFD	0.343	9.639	0.000
PHB→AWFD	0.078	6.283	0.000
AOC→PNS	0.133	3.801	0.000
AOC→IWFD	0.070	3.029	0.001
AOC→AWFD	0.016	2.897	0.002
AOR→IWFD	0.120	3.917	0.000
AOR→AWFD	0.027	3.560	0.000
PNS→AWFD	0.137	6.732	0.000
SNS→AWFD	0.052	3.661	0.000

**Note:** HV: Health Values, HC: Health Consciousness, HKS: Health Knowledge-Seeking, PHB: Personal Health Beliefs, AOC: Awareness of Consequences, AOR: Ascription of Responsibility, PNS: Personal Norms; SNS: Social Norms, IWFD: Intention to Adopt IoT-enabled WFD, AWFD: Actual Adoption of IoT-enabled WFD.

## 5 Discussion

The current study used the VBN model to examine the influence of health values, health consciousness, and health knowledge-seeking on personal health beliefs. Next, this study assessed the relationships between personal norms with health beliefs, awareness of consequences, and ascription of responsibility. The relationships between the intention to adopt IoT-enabled WFDs with personal norms and social norms, as well as the relationship between the intention to adopt IoT-enabled WFDs and the actual adoption of IoT-enabled WFDs, were thoroughly assessed. Furthermore, all indirect effects were evaluated according to the proposed research framework. The following section presents the main findings of the study.

First, the health values in this study did not exhibit any considerable impact on personal health beliefs (H1). However, Lee and Lee [29] and Hossain et al. [34] reported different result. This finding suggests the importance of health values in the adoption of wearable healthcare devices. One possible reason for such outcome is because young adults are so preoccupied with other activities that they do not prioritize their fitness over these other activities. In other words, they believe that many other aspects of life can make them happy. Following that, health consciousness in this study exhibited significant influence on personal health beliefs (H2). Rupp et al. [44] and Hossain et al. [34] reported similar findings. In other words, individuals have become more health-conscious and placed more emphasis of becoming more active and fitter. The participating young adults in this study evaluated fitness monitoring systems for enhanced health support advantages to prevent critical illnesses. Although the findings from H1 and H2 may initially seem contradictory, a more careful analysis reveals complementary insights that contribute to a nuanced understanding of the factors affecting personal health beliefs in the context of WFDs. Rejection of H1 illuminates the disparity between individuals’ health values and their actual health beliefs and behaviours, highlighting the influence of competing priorities and time constraints on health-related decision-making. On the other hand, the acceptance of H2 indicates a strengthening trend towards health consciousness and awareness, suggesting a positive shift towards prioritizing health and fitness despite the obstacles posed by other obligations. Additionally, health knowledge-seeking in this study was found to have substantial and favourable influence on personal health beliefs (H3). This particular finding is congruent with the finding of earlier studies [1]. This implies that young adults who plan to pursue self-care are more eager to learn about the right health fitness criteria, promoting strong personal health beliefs to adopt IoT-enabled WFDs for fitness tracking.

Based on the results, personal health beliefs had significant and positive influence on awareness of consequences (H4a) and personal norms (H4b). These findings are in line with the findings of prior studies on wearable healthcare devices [28,32]. This confirms that IoT-enabled WFDs are appreciated for continuous fitness monitoring. These health beliefs may indeed drive young adults to become more aware of the negative repercussions of chronic diseases and related treatment expenses. Moreover, the findings also indicated that awareness of consequences had a significant impact on ascription of responsibility (H5a). In addition, both awareness of consequences (H5b) and ascription of responsibility (H6) were found to have major influence on personal norms. Focusing on the COVID-19 prevention measures, Shanka and Gebremariam [24] reported similar findings. This proves that individuals who are aware of the negative ramifications of unfit and physically inactive lifestyle are more likely to believe that they are morally obligated and instinctively accountable to assess their physical fitness, ensuring that they perform adequate fitness activities.

Another notable outcome of this study was the significant influence of personal norms (H7) and social norms (H8) on the intention to adopt IoT-enabled WFDs. Previous studies that explored the contexts of IoT-healthcare and mobile-healthcare [29,38], corroborated these findings. In other words, individuals who realise their moral accountability for their own physical fitness, as well as those who are exposed to the adverse consequences of chronic illnesses due to their unfit physical conditions are more likely to demonstrate strong intention to adopt IoT-enabled WFDs. Likewise, the results demonstrated the major role of social norms in enhancing one's inclination to embrace IoT-enabled WFDs, as young adults tend to follow societal trends and are inspired by the physically fit prominent figures around them. Young adults who witness how other individuals use IoT-enabled WFDs are more likely to be motivated to track their fitness using such gadgets.

Finally, this study demonstrated that the intention to adopt IoT-enabled WFDs strongly and favourably affected their actual adoption (H9). This finding is consistent with Princi and Krämer [38] empirical findings within the context of IoT-healthcare devices. These findings can be interpreted as follows: individuals with strong intention to adopt IoT-enabled WFDs are more likely to be the apparent users of IoT-based WFDs. All extrinsic and intrinsic healthy lives and physical fitness-related values, beliefs, and norms play influential roles in users’ decision-making of using IoT-enabled WFDs.

### 5.1 Implications

#### 5.1.1 Theoretical implications

This study examined an underexploited theoretical foundation for users' decision-making processes in the adoption of IoT-enabled WFDs and developed a grounded theory that contributes to the current literature on technology adoption behaviour. To fully understand the consequences of fitness monitoring for the maintenance of physically active lifestyle, this study expanded the VBN model by incorporating four relatively untapped aspects, specifically health values, health consciousness, health knowledge-seeking, and personal health beliefs, within the context of adoption of IoT-enabled WFDs. Furthermore, the integration of additional constructs, such as health knowledge-seeking broadens the theoretical framework and deepens our understanding of the intricate interplay among individual beliefs, social norms, and behavioral intentions. Moreover, the empirical evaluation of the proposed expanded VBN model provides valuable theoretical insights into the existing literature on the adoption of IoT-enabled WFDs among young adults in developing countries. Based on the obtained results, the model's proposed components, except health values, exhibited significant and strong influence on the adoption of IoT-enabled WFDs, confirming the high explanatory power of the model. Most prior studies have concentrated on the technical aspects of IoT-enabled gadgets and their applications, but the current study broadened the knowledge by conceptualising both intrinsic and extrinsic elements of IoT-enabled device adoption, with the goal of understanding users' decision-making processes within the context of physical fitness monitoring.

#### 5.1.2 Practical and managerial implications

Based on the findings, this study presented several practical and managerial implications that guide marketers, practitioners, and policymakers in the health monitoring sector to efficiently plan marketing components that favour IoT-enabled device adoption. Based on the obtained results, personal health beliefs for adopting IoT-enabled WFDs were found to be influenced by health consciousness and health knowledge-seeking. Considering that, marketers should carefully communicate the practical benefits of fitness monitoring using IoT-based devices via strategic promotional efforts. Practitioners may enrich users with customised information and health knowledge, such as appropriate physical exercise moves based on body mass, suitable schedule and duration for workout, and information on the complexities of obesity, to emphasise the sophisticated characteristics of IoT-enabled WFDs. Practitioners may also address certain user groups to manifest a sense of fitness improvement and confidence in them, while also enhancing their awareness on the benefits of using IoT-enabled WFDs daily for fitness tracking. Concurrently, the government's public health department may take efforts to equip the community with additional health and fitness-related information, boosting their moral beliefs for a healthy and sickness-free future.

Personal health beliefs were found to have significant impact on personal norms and awareness of consequences. Developers are encouraged to incorporate personalized health features into their platforms. By utilizing the data-driven insights gleaned from our study, they can craft health-related recommendations and interventions customized to the unique preferences, behaviours, and health status of individuals. The inclusion of personalization can significantly boost user engagement and enhance the efficacy of health-promotion initiatives. Particularly, developers may incorporate a few unique features in the development of IoT-based WFDs, such as features for early detection of critical illnesses, sleep, and diet monitoring, reporting of any irregularities, and workload evaluation and monitoring. Moreover, developers should consider integrating features such as real-time health tracking, activity monitoring, and personalized health dashboards. In addition, daily workout plan and record keeping features may benefit and motivate users to form a regular health fitness assessment and sensibly embrace IoT-enabled WFDs. Furthermore, maintaining consistency in the updates of middleware and ensuring to innovate with distinguishing features may increase users' reliance on IoT-enabled WFDs and their services, which subsequently generate strong bond among users. Adhering to strict quality standards is a vital component for IoT brands to obtain and retain users' trust in IoT-enabled WFDs. Concurrently, the government's public health department should strategically launch educational programmes and public awareness campaigns to promote the benefits of physical exercise, diet maintenance, and fitness tracking using IoT-enabled WFDs, motivating the public to be proactive in preventing early chronic medical conditions.

Realising the significance of social norms in determining one's intention to adopt IoT-enabled WFDs, knowledge regarding the products alone may not be enough to persuade all users to consider adopting the devices if they cannot recognize social endorsement over the claimed advantages. Advertisers and practitioners should publish appropriate information about the integrated features and services of IoT-enabled WFDs to guarantee that their claims of fitness monitoring advantages are justified. Moreover, IoT-enabled WFDs can be widely adopted if well-known personalities, celebrities, and real-life heroes recommend and endorse using such technologies. Marketers may introduce different offers and discounts to early adopters who may serve as real-life evidence and references, spreading positive word-of-mouth and wide-scale acceptability of IoT-enabled WFDs. Additionally, IoT brands may consider engaging different corporate social responsibilities and sponsoring different social-sports communities to raise public awareness of fitness and healthy life. Finally, considering the growing recognition of mental health issues, it is essential that developers prioritize incorporating features that promote mental well-being. Examples of such features include mindfulness practices, resources for stress management, and tools that foster a positive online atmosphere. A comprehensive approach to health promotion can be achieved by leveraging technology to address mental-health concerns. We also found a positive effect of health knowledge-seeking on health beliefs, emphasizing the pivotal role of information acquisition and dissemination in shaping individuals' beliefs, attitudes, and intentions towards WFDs. These findings highlight the transformative power of wearable devices as platforms for health education, promoting awareness of preventive measures, and fostering continuous learning and self-improvement.

Finally, industry professionals, manufacturers and developers can use our insights to fine-tune their marketing strategies and product development efforts. By highlighting the health advantages, convenience, and user experience provided by IoT-enabled WFDs, companies can effectively target and attract consumers who are looking to improve their fitness and wellness. Furthermore, healthcare facilities can explore the possibilities of integrating IoT-enabled WFDs into their patient care programs and wellness initiatives. Healthcare providers can use the data generated by these devices to customize treatment plans and promote preventive health measures. IT developers can be creative and can develop new applications, features, and services that enhance the functionality and usability of IoT-enabled WFDs. By incorporating cutting-edge technologies, such as artificial intelligence and data analytics, developers can improve the accuracy of health data monitoring, provide personalized insights, and increase user engagement with these devices.

#### 5.1.3 Limitations and future research Directions

This study has several limitations that should be addressed in future research. First, the data were gathered from a small sample of IoT-enabled device users in China, which may restrict the model's generalizability in other scenarios. It is recommended for future research to collect and analyse data from a larger sample with diverse demographic characteristics from different locations to gain better insights on the adoption of IoT-enabled healthcare devices. Second, we collected data from young adults aged 18–30 years. Focusing on this age group allowed us to gain useful insights into the adoption behaviours and preferences of younger adults; however, it did not consider the viewpoints, behaviours, and motivations of older adults or those outside of this age range, which reduced the ability to apply our findings to a wider range of ages. Therefore, future researchers should include a broader range of age groups to gain a comprehensive understanding of adoption patterns among different demographic groups. Third, the current study only focused on a few components of values, beliefs, and norms, and it is evident that there are additional key factors that may have been underexplored, such as technology acceptance factors. Therefore, future research should consider more factors or elements to improve existing knowledge. Fourth, this study thoroughly investigates the “value” aspect of individuals' evaluations of IoT-enabled WFDs, recognizing that specific value-related concepts, such as convenience, data-driven choices, lifestyle improvements, hedonic enjoyment, and symbolic value, were not directly included in the analysis. To gain a more comprehensive understanding, future research could use mixed-method approaches that encompass a wider range of value-related factors and their impact on promoting the adoption and utilization of WFDs among various user groups. Fifth, this study employed a cross-sectional quantitative method, which limited the investigation of behaviour across time. Considering this, future research should use a longitudinal approach to examine the long-term consequences of the model's components and their relevant associations across time. The sixth point acknowledges the possibility of gender bias due to the high proportion of female participants constituting 67 % of the sample, which may impact the generalizability of our study outcomes. To ensure the robustness and validity of the study results across diverse user populations, future research should aim to achieve more balanced gender representation. Finally, it is crucial to recognize that our study relied on self-selected respondents for data collection, which may have introduced biases associated with volunteer or convenience sampling methods. Future studies should explore alternative sampling approaches to enhance representativeness and minimize potential biases related to self-selection.

## 6 Conclusion

Wearable fitness monitors have become mainstream of the health and fitness tracking methods as the low levels of physical activities among the younger generations increase the risks of obesity and unhealthy lifestyle. Focusing on young adults in China, this study identified significant factors that influence the decision to adopt IoT-enabled WFDs, which have beneficial impact on physical activity behaviours, body mass measurements, and overall perceptions of health and well-being. Using the extended VBN model, this study empirically examined the core values, beliefs, and norms associated with features that motivate users to adopt IoT-enabled WFDs to monitor their fitness and daily physical activities. Furthermore, the current study addressed significant gaps in literature by examining the socio-psychological factors that influence users' decision to embrace IoT-enabled WFDs. This study demonstrated the substantial influence of health consciousness and health knowledge-seeking on personal health beliefs, which positively drive personal norms and awareness of consequences. Awareness of consequences fosters ascription of responsibility, which influences personal norms. Likewise, social norms play a substantial role in positively influencing the young adults' intention to adopt IoT-enabled WFDs. This study's findings highlight the need for developers and manufacturers to incorporate users' trendy behavioural aspects and design distinctive features into their products to meet a diverse range of market demands. The findings suggest that developers and public health organizations should devise tailored health-related recommendations and interventions that cater to the specific preferences, behaviours, and health conditions of individuals. Additionally, the incorporation of features such as real-time health monitoring, activity tracking, and individualized health dashboards can further enhance the effectiveness of these recommendations and interventions. Furthermore, the use of fitness trackers in conjunction with wellness education, which potentially alters the younger generations' beliefs and expectations, can be an effective approach to initiate behavioural changes. Therefore, this study suggests that government's public health department and market practitioners should develop appropriate strategies.

## Ethical approval

The research ethics committee of Changzhi University, China have approved this study (Approval Number: CZ-2022-0079). This study has been performed in accordance with the Declaration of Helsinki.

## Informed consent

Written informed consent for participation was obtained from respondents who participated in the survey. For the respondents who participated in the survey were asked to read the ethical statement posted at the top of the form and proceed only if they agree.

## Conflicting interests

The authors declare that the research was conducted in the absence of any commercial or financial relationships that could be construed as a potential conflict of interest.

## Funding

This research received no specific grant from any funding agency in the public, commercial, or not-for-profit sectors.

## Authorship

Qing Yang and Farzana Naznen: Conceptualization, Methodology, Writing - Original Draft. Abdullah Al Mamun and Mohammad Nurul Hassan Reza: Conceptualization, Methodology, Formal Analysis, Methodology, Writing - Review & Editing.

## Data availability statement

Data included in article/supp. material/referenced in article.

## CRediT authorship contribution statement

**Qing Yang:** Writing – original draft, Methodology, Data curation, Conceptualization. **Abdullah Al Mamun:** Writing – review & editing, Investigation, Formal analysis, Conceptualization. **Mohammad Nurul Hassan Reza:** Writing – review & editing, Methodology, Conceptualization. **Farzana Naznen:** Writing – original draft, Methodology, Conceptualization.

## Declaration of competing interest

The authors declare the following financial interests/personal relationships which may be considered as potential competing interests: This manuscript's corresponding author (Abdullah Al Mamun) is also an Associate Editor at Heliyon (Business and Management). If there are other authors, they declare that they have no known competing financial interests or personal relationships that could have appeared to influence the work reported in this paper.

## References

[1] Kim B., Hong S., Kim S. (2021). Introducing an integrated model of adults' wearable activity tracker Use and obesity information–seeking behaviors from a National Quota sample survey. JMIR Formative Research.

[2] Yacchirema D., de Puga J.S., Palau C., Esteve M. (2019). Fall detection system for elderly people using IoT and ensemble machine learning algorithm. Personal Ubiquitous Comput..

[3] Centers for Disease Control & Prevention (2022). Adult Obesity Facts.

[4] Kristoffersson A., Lindén M. (2020). A systematic review on the Use of wearable body sensors for health monitoring: a Qualitative Synthesis. Sensors.

[5] Yang Q., Al Mamun A., Hayat N., Jingzu G., Hoque M.E., Salameh A.A. (2022). Modeling the intention and adoption of wearable fitness devices: a study using SEM-PLS analysis. Front. Public Health.

[6] Clubbs B.H., Gray N., Madlock P. (2021). Using the theory of planned behavior and the technology acceptance model to analyze a university employee fitness tracker program with financial incentive. J. Commun. Healthc..

[7] Ganji K., Parimi S. (2022). ANN model for users' perception on IOT based smart healthcare monitoring devices and its impact with the effect of COVID 19. Journal of Science and Technology Policy Management.

[8] Pataranutaporn P., Jain A., Johnson C.M., Shah P., Maes P. (2019, 23-27 July 2019). 2019 41st Annual International Conference of the IEEE Engineering in Medicine and Biology Society (EMBC).

[9] Kao Y.-S., Nawata K., Huang C.-Y. (2019). An exploration and Confirmation of the factors influencing adoption of IoT-based wearable fitness trackers. Int. J. Environ. Res. Publ. Health.

[10] Owens J., Cribb A. (2019). ‘My Fitbit thinks I can do better!’ Do health promoting wearable technologies support personal Autonomy?. Philosophy & Technology.

[11] Swan M. (2012). Sensor Mania! The internet of things, wearable computing, objective metrics, and the Quantified self 2.0. J. Sens. Actuator Netw..

[12] Yang Q., Al Mamun A., Hayat N., Md Salleh M.F., Salameh A.A., Makhbul Z.K.M. (2022). Predicting the mass adoption of eDoctor apps during COVID-19 in China using hybrid SEM-neural network analysis. Front. Public Health.

[13] UNCTAD (2022). https://unctad.org/system/files/official-document/der2021_en.pdf.

[14] Loucks Jeff, Stewart Duncan, Bucaille Ariane, Crossan G. (2022). https://www2.deloitte.com/us/en/insights/industry/technology/technology-media-and-telecom-predictions/2022/wearable-technology-healthcare.html.

[15] Statista (2023). Market size of smart wearables in China 2017-2023. https://www.statista.com/statistics/1287657/china-smart-wearables-market-size/.

[16] Statista (2024). Smartwatches - China. https://www.statista.com/outlook/hmo/digital-health/digital-fitness-well-being/fitness-trackers/smartwatches/china#:%7E:text=The%20Smartwatches%20market%20is%20set,US%2418.95bn%20in%202024.

[17] SCIO (2020). Status of Nutrition and chronic diseases of the Chinese population. http://english.scio.gov.cn/pressroom/2020-12/23/content_77043604.htm.

[18] Schaben J.A., Furness S. (2018). Investing in college students: the role of the fitness tracker. DIGITAL HEALTH.

[19] Indrakumari R., Poongodi T., Suresh P., Balamurugan B., Raj P., Evangeline P. (2020).

[20] Sucharitha G., Tannmayee B., Dwarakamai K., Nandan Mohanty S., Chatterjee J.M., Satpathy S. (2022). Internet of Things and its Applications.

[21] Verma D., Singh K.R.B., Yadav A.K., Nayak V., Singh J., Solanki P.R., Singh R.P. (2022). Internet of things (IoT) in nano-integrated wearable biosensor devices for healthcare applications. Biosens. Bioelectron. X.

[22] Boudreau F., Godin G. (2014). Participation in regular Leisure-time physical activity among individuals with type 2 diabetes not meeting Canadian guidelines: the influence of intention, perceived behavioral control, and moral norm. Int. J. Behav. Med..

[23] McBride C.M., Morrissey E.C., Molloy G.J. (2020). Patients' experiences of using smartphone apps to support self-management and improve Medication Adherence in hypertension: Qualitative study. JMIR Mhealth Uhealth.

[24] Shanka M.S., Gebremariam K.M. (2023). Combining rationality with morality – integrating theory of planned behavior with norm activation theory to explain compliance with COVID-19 prevention guidelines. Psychol. Health Med..

[25] Ajzen I. (2020). The theory of planned behavior: Frequently asked questions. Human Behavior and Emerging Technologies.

[26] Cheng E.W.L. (2019). Choosing between the theory of planned behavior (TPB) and the technology acceptance model (TAM). Educ. Technol. Res. Dev..

[27] Beh P.K., Ganesan Y., Iranmanesh M., Foroughi B. (2021). Using smartwatches for fitness and health monitoring: the UTAUT2 combined with threat appraisal as moderators. Behav. Inf. Technol..

[28] Cheung M.L., Chau K.Y., Lam M.H., Tse G., Ho K.Y., Flint S.W., Broom D.R., Tso E.K., Lee K.Y. (2019). Examining consumers' adoption of wearable healthcare technology: the role of health attributes. Int. J. Environ. Res. Publ. Health.

[29] Lee S.M., Lee D. (2020). Healthcare wearable devices: an analysis of key factors for continuous use intention. Service Business.

[30] Sadeck O. (2022).

[31] Marikyan D., Papagiannidis S. (2023). http://open.ncl.ac.uk.

[32] Zhang M., Luo M., Nie R., Zhang Y. (2017). Technical attributes, health attribute, consumer attributes and their roles in adoption intention of healthcare wearable technology. Int. J. Med. Inf..

[33] Stern P.C., Dietz T., Abel T., Guagnano G.A., Kalof L. (1999). A value-belief-norm theory of support for social Movements: the case of Environmentalism. Hum. Ecol. Rev..

[34] Hossain M.I., Yusof A.F., Hussin A.R.C., lahad N.A., Sadiq A.S. (2021). Factors influencing adoption model of continuous glucose monitoring devices for internet of things healthcare. Internet of Things.

[35] Putri K., Abdullah Z., Raza S.H., Istiyanto S.B. (2021). The antecedents and consequences of health information seeking and behavioral intention. Journal of Management Information and Decision Sciences.

[36] Pu B., Zhang L., Tang Z., Qiu Y. (2020). The relationship between health consciousness and Home-based exercise in China during the COVID-19 pandemic. Int. J. Environ. Res. Publ. Health.

[37] Sharon T. (2017). Self-tracking for health and the Quantified self: Re-Articulating Autonomy, Solidarity, and Authenticity in an age of personalized healthcare. Philosophy & Technology.

[38] Princi E., Krämer N.C. (2020). Out of control–privacy calculus and the effect of perceived control and moral considerations on the usage of IoT healthcare devices. Front. Psychol..

[39] Kushwah S., Dhir A., Sagar M., Gupta B. (2019). Determinants of organic food consumption. A systematic literature review on motives and barriers. Appetite.

[40] Lau R.R., Hartman K.A., Ware J.E. (1986). Health as a value: methodological and theoretical considerations. Health Psychol..

[41] Piko B.F., Keresztes N. (2006). Physical activity, psychosocial health and life goals among youth. J. Community Health.

[42] Feng S., Mäntymäki M., Dhir A., Salmela H. (2021). How self-tracking and the Quantified self promote health and well-being: systematic review. J. Med. Internet Res..

[43] Hsu S.-Y., Chang C.-C., Lin T.T. (2016). An analysis of purchase intentions toward organic food on health consciousness and food safety with/under structural equation modeling. Br. Food J..

[44] Rupp M.A., Michaelis J.R., McConnell D.S., Smither J.A. (2018). The role of individual differences on perceptions of wearable fitness device trust, usability, and motivational impact. Appl. Ergon..

[45] Li H., Gupta A., Zhang J., Sarathy R. (2014). Examining the decision to use standalone personal health record systems as a trust-enabled fair social contract. Decis. Support Syst..

[46] Ataei P., Gholamrezai S., Movahedi R., Aliabadi V. (2021). An analysis of farmers' intention to use green pesticides: the application of the extended theory of planned behavior and health belief model. J. Rural Stud..

[47] Klöckner C.A., Ohms S. (2009). The importance of personal norms for purchasing organic milk. Br. Food J..

[48] Nguyen T.P.L., Doan X.H., Nguyen T.T., Nguyen T.M. (2021). Factors affecting Vietnamese farmers' intention toward organic agricultural production. Int. J. Soc. Econ..

[49] Han H., Olya H.G., Kim J., Kim W. (2018). Model of sustainable behavior: Assessing cognitive, emotional and normative influence in the cruise context. Bus. Strat. Environ..

[50] Kwon O., Bae S., Shin B. (2020). Understanding the adoption intention of AI through the ethics lens. http://hdl.handle.net/10125/64353.

[51] Arnold T., Scheutz M. (2018). The “big red button” is too late: an alternative model for the ethical evaluation of AI systems. Ethics Inf. Technol..

[52] Ulijaszek S.J. (2017).

[53] Reith R., Buck C., Eymann T., Lis B. (2020). Integrating privacy concerns into the unified theory of acceptance and use of technology to explain the adoption of fitness trackers. Int. J. Innovat. Technol. Manag..

[54] Ghazali E.M., Nguyen B., Mutum D.S., Yap S.-F. (2019). Pro-environmental behaviours and value-belief-norm theory: Assessing Unobserved Heterogeneity of Two Ethnic groups. Sustainability.

[55] Rahmatipour M.A., Ebadollahi-Natanzi A., Arab-Rahmatipour G. (2020). Prevention of depression and psychological stress by studying book in quarantine conditions of COVID-19. SciMedicine Journal.

[56] Ahmed W., Hizam S.M., Sentosa I., Akter H., Yafi E., Ali J. (2020). Predicting IoT service adoption towards smart mobility in Malaysia: SEM-neural hybrid pilot study. Int. J. Adv. Comput. Sci. Appl..

[57] Hair J.F., Hult G.T.M., Ringle C.M., Sarstedt M. (2021).

[58] Hair Jr J.F., Hult G.T.M., Ringle C.M., Sarstedt M., Danks N.P., Ray S. (2021).

[59] Bornstein M.H., Jager J., Putnick D.L. (2013). Sampling in developmental science: Situations, shortcomings, solutions, and standards. Dev. Rev..

[60] Winton B.G., Sabol M.A. (2022). A multi-group analysis of convenience samples: free, cheap, friendly, and fancy sources. Int. J. Soc. Res. Methodol..

[61] Doebel, S., & Frank, M. C. Broadening Convenience Samples to Advance Theoretical Progress and Avoid Bias in Developmental Science. J. Cognit. Dev., 1-12. 10.1080/15248372.2023.2270055.

[62] Faul F., Erdfelder E., Buchner A., Lang A.-G. (2009). Statistical power analyses using G*Power 3.1: Tests for correlation and regression analyses. Behav. Res. Methods.

[63] Bujang M.A., Sa'at N., Sidik T., Joo L.C. (2018). Sample size guidelines for logistic regression from Observational studies with large population: emphasis on the accuracy between Statistics and parameters based on real life Clinical data. Malays. J. Med. Sci..

[64] Hong H. (2009).

[65] Kraft F.B., Goodell P.W. (1993). Identifying the health conscious consumer. J. Health Care Market..

[66] Yang Q., Al Mamun A., Naznen F., Siyu L., Mohamed Makhbul Z.K. (2023). Modelling the significance of health values, beliefs and norms on the intention to consume and the consumption of organic foods. Heliyon.

[67] Doran R., Larsen S. (2016). The relative importance of social and personal norms in explaining intentions to choose eco‐friendly travel options. Int. J. Tourism Res..

[68] Alam M.Z., Hoque M.R., Hu W., Barua Z. (2020). Factors influencing the adoption of mHealth services in a developing country: a patient-centric study. Int. J. Inf. Manag..

[69] Wang H., Tao D., Yu N., Qu X. (2020). Understanding consumer acceptance of healthcare wearable devices: an integrated model of UTAUT and TTF. Int. J. Med. Inf..

[70] Change S., Witteloostuijn A., Eden L. (2010). From the editors: common method variance in international research. J. Int. Bus. Stud..

[71] Podsakoff P.M., MacKenzie S.B., Podsakoff N.P. (2012). Sources of method bias in social science research and recommendations on how to control it. Annu. Rev. Psychol..

[72] Kock N. (2015). Common method bias in PLS-SEM: a full collinearity assessment approach. Int. J. e-Collaboration.

[73] Hair J.F., Risher J.J., Sarstedt M., Ringle C.M. (2019). When to use and how to report the results of PLS-SEM. Eur. Bus. Rev..

[74] Henseler J., Sarstedt M. (2013). Goodness-of-fit indices for partial least squares path modeling. Comput. Stat..

[75] Cohen J. (2013).

