# Supplementary material for: Modelling the significance of value-belief-norm framework to predict mass adoption potentials of internet of things-enabled wearable fitness devices
Source: Heliyon. 2024 Apr 28;10(9):e30179. doi: 10.1016/j.heliyon.2024.e30179 (PMC11088247; doi:10.1016/j.heliyon.2024.e30179)
Supplement: Multimedia component 1 [file mmc1.docx]

**Table S1.** Survey Instrument.

| HV1 | If I don’t have my health, I don’t have anything |
| --- | --- |
| HV2 | There is nothing I care more about than my health |
| HV3 | Good health is most important for happy life |
| HV4 | Nothing is more important than good health |
| HC1 | I think my health depends on how well I take care of myself |
| HC2 | I am actively engaged in the prevention of disease and illness |
| HC3 | I think taking preventive measures help to stay healthy |
| HC4 | Living a healthy life is important to me |
| HC5 | I am constantly watchful about my health |
| HKS1 | I often read about health-related discussion in print media. |
| HKS2 | I often search for health information on the internet. |
| HKS3 | I try to find information on the internet whenever I notice unusual symptoms. |
| HKS4 | I regularly talk with my family doctor. |
| HKS5 | I try to find updated knowledge about new kinds of illness and disease. |
| PHB1 | It is not the doctors’ job to keep me well |
| PHB2 | My health is in my control. |
| PHB3 | I think that the idea of ‘wellness’ is not a fad. |
| PHB4 | It is the government’s job to keep me well. |
| PHB5 | I only worry about my health even when I am not sick. |
| AOC1 | Personal health management can improve the individual quality of life. |
| AOC2 | Adaptation of exercise plan can reduce the negative consequences towards personal health. |
| AOC3 | Health issues can be curtailed with the management of personal health. |
| AOC4 | Minor health concerns may lead to grave health issues. |
| AOC5 | Overall, personal management of health can improve personal health. |
| AOR1 | I think taking responsibility for personal health is important. |
| AOR2 | I feel that taking responsibility for personal healthcare can help to promote a healthy society. |
| AOR3 | Taking personal responsibility for healthcare to promote personal wellbeing. |
| AOR4 | Everyone must take responsibility for personal health. |
| AOR5 | I feel responsible for personal healthcare. |
| PNS1 | I feel ethically responsible for reducing the burden on the hospitals by monitoring my health using IOT enabled Wearable Fitness Devices |
| PNS2 | People like me should use IOT enabled Wearable Fitness Devices to reduce the burden on the healthcare system. |
| PNS3 | I feel morally obliged to use IOT enabled Wearable Fitness Devices, although it might be more expensive. |
| PN4 | I morally think that using IOT enabled Wearable Fitness Devices are essential, regardless of what others do. |
| PCN5 | I feel personally feel obliged to use IOT enabled Wearable Fitness Devices. |
| SNS1 | Most of the people in my companionship use IOT enabled Wearable Fitness Devices. |
| SNS2 | Most people in my life are making efforts to use the IOT enabled Wearable Fitness Devices. |
| SNS3 | Most of my peers willingly engage in using IOT enabled Wearable Fitness Devices |
| SNS4 | Most of my peers enthusiastic about the benefits of IOT enabled Wearable Fitness Devices. |
| SNS5 | Most of my peers enthusiastic about the price value of IOT enabled Wearable Fitness Devices. |
| UIM1 | I intend to use IOT enabled Wearable Fitness Devices to manage my health in the future. |
| UIM2 | I will always try to use IOT enabled Wearable Fitness Devices to manage my health in my daily life in the future. |
| UIM3 | I plan to use IOT enabled Wearable Fitness Devices frequently to manage my health in the future. |
| UIM4 | I would be willing to develop a habit to use IOT enabled Wearable Fitness Devices every day in the future. |
| UIM5 | I predict I will use IOT enabled Wearable Fitness Devices to manage my health information. |
| AWFD1 | How often do you use IOT enabled Wearable Fitness Devices? (Never – Rarely – Sometimes – Often – Always) |


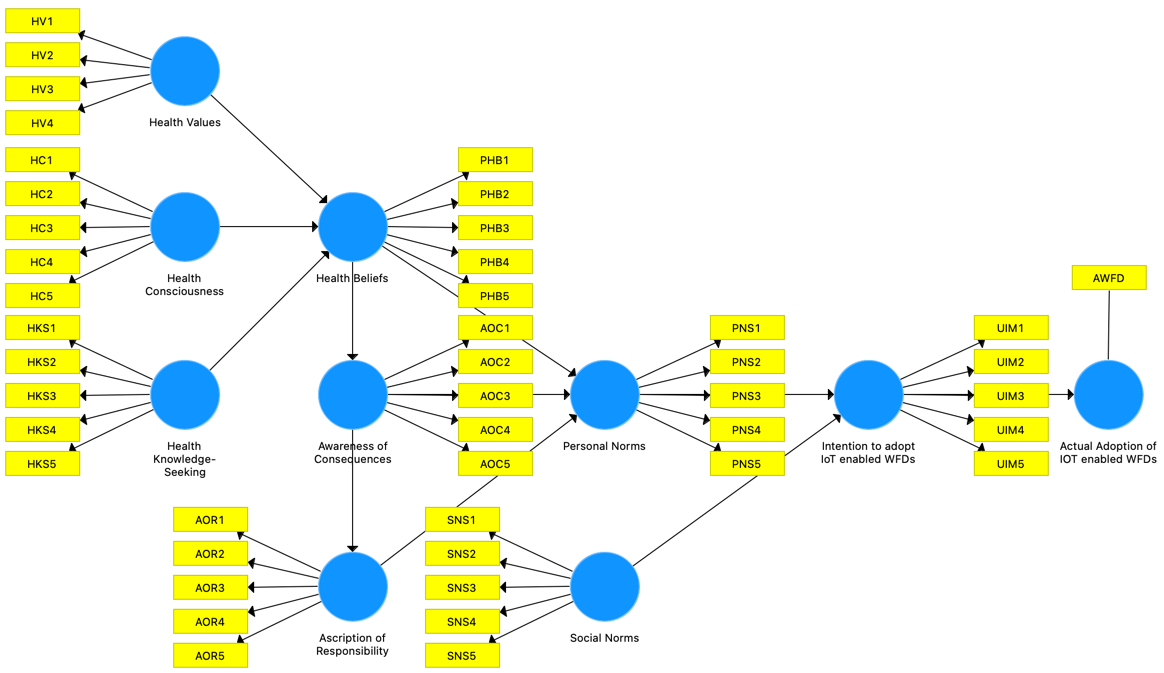


**Figure S1.** PLS-SEM Model
